# Protocol for detecting SnRK2 kinase activity in plants by immunoblotting, in-gel assay, band shift, and immunoprecipitation

**DOI:** 10.1016/j.xpro.2025.103842

**Published:** 2025-05-28

**Authors:** Qingzhong Li, Xianping Yuan, Yang Zhao

**Affiliations:** 1 Key Laboratory of Plant Carbon Capture, Shanghai Center for Plant Stress Biology, CAS Center for Excellence in Molecular Plant Sciences, Chinese Academy of Sciences, Shanghai 200032, China; 2 University of Chinese Academy of Sciences, Beijing 100049, China

**Keywords:** Model Organisms, Plant sciences, Protein Biochemistry

## Abstract

The SNF1-regulated protein kinase 2s (SnRK2s) are activated by phytohormone abscisic acid (ABA) and osmotic stress to control plant growth and stress responses; however, assessing SnRK2 activity is challenging. Here, we present a protocol to detect SnRK2 activity in plants. We describe steps for performing immunoblotting with anti-phospho-S175-SnRK2 antibody, in-gel kinase assay, band-shift assay, and immunoprecipitated kinase assay. Immunoblotting and in-gel kinase assays are suitable for evaluating endogenous SnRK2 activity, whereas band-shift and immunoprecipitated kinase assays are applicable to assess tagged SnRK2 activity in transgenic lines.

For complete details on the use and execution of this protocol, please refer to Li et al.,^1^ Li et al.,^2^ and Yuan et al.^3^

## Highlights

• Instructions for evaluating plant SnRK2 kinase activity *in vivo*
• Steps for monitoring SnRK2 activation through immunoblotting and in-gel kinase assays
• Applications for both endogenous and tagged SnRK2 variants

## Abstract (editor highlights)

Publisher’s note: Undertaking any experimental protocol requires adherence to local institutional guidelines for laboratory safety and ethics.

## Graphical abstract

**Figure:**
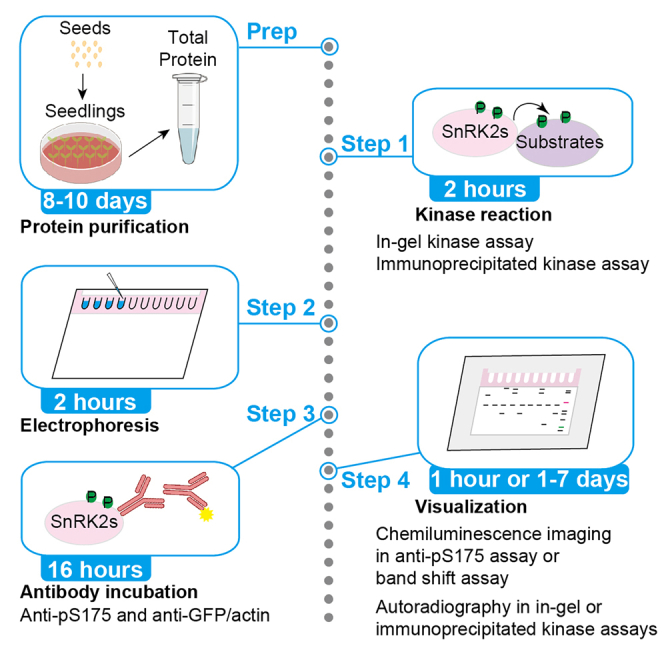

## Before you begin

SnRK2s are evolutionarily conserved core protein kinases controlling hyperosmotic stress and ABA signaling in plants.^4^ They are activated by the phytohormone ABA, and by hyperosmotic stresses triggered by multiple stimuli, including dehydration,^1^ cold,^5^ salinity, and high concentrations of mannitol, sorbitol, and other solutes (e.g., 300–800 mM).^2^^,^^3^ However, assessing SnRK2 activity via the classical in-gel kinase assay is challenging, limiting our understanding of osmotic stress sensing, SnRK2 activation, and signaling crosstalk with other stimuli. We generated the anti-phospho-S175-SnRK2s antibody, which recognizes the phosphorylation of SnRK2.6 (also named OST1 or SRK2E) at Ser175 and the phosphorylation of serine residues in the activation loop of multiple SnRK2s corresponding to Ser175 in SnRK2.6.^3^^,^^6^^,^^7^^,^^8^^,^^9^^,^^10^^,^^11^^,^^12^ The phosphorylation level of this conserved serine reflects SnRK2 activity. Therefore, immunoblotting with the anti-phospho-S175-SnRK2s antibody can be used as a substitute for the classical SnRK2 in-gel kinase assay, breaking down the barriers for studying SnRK2 activation and early osmotic stress signaling. SnRK2 activity can be further evaluated by detecting the phosphorylation of substrates (e.g., histones and ABF2 fragment) by SnRK2s via the in-gel kinase assays. The immunoblotting and in-gel kinase assays are suitable for evaluating endogenous SnRK2 activities in various mutants and transgenic lines.^1^^,^^2^ SnRK2s belong to a large family and are differentially activated by ABA and milder or severe osmotic stresses.^3^ Using transgenic lines, the band shift and immunoprecipitated kinase assays help us to assess the activity of specific members of SnRK2s, uncovering their unique regulatory mechanisms.^3^ The *SnRK2.1pro:SnRK2.1-GFP* and *SnRK2.4pro:SnRK2.4-GFP* transgenic lines in *snrk2.1/4/5/7/8/9/10* mutant background, but not the overexpression lines, are suitable for band shift assays.^3^ These four methods will facilitate the evaluation of SnRK2 kinase activity in plants.

## Key resources table

**Table:** 

REAGENT or RESOURCE	SOURCE	IDENTIFIER
**Chemicals, peptides, and recombinant proteins**		

Sodium hypochlorite (NaClO)	Sinopharm Chemical Reagent	CAS#: 7681-52-9
75% alcohol	General reagent	CAS#: G73537W
Murashige & Skoog (MS) basal salt mixture	Phyto Technology	Cat#: M524
Sucrose	Sinopharm Chemical Reagent	CAS#: 57-50-1
Agar	Sigma-Aldrich	CAS#: 9002-18-0
NaCl	Sinopharm Chemical Reagent	CAS#: 7647-14-5
ABA	Sinopharm Chemical Reagent	CAS#: 14375-45-2
Mannitol	Sinopharm Chemical Reagent	CAS#: 69-65-8
Tris	Sinopharm Chemical Reagent	CAS#: 77-86-1
Sodium dodecyl sulfate (SDS)	Sigma-Aldrich	CAS#: 151-21-3
Glycine	Sigma-Aldrich	CAS#: 56-40-6
HEPES	Sigma-Aldrich	CAS#: 7365-45-9
EDTA	Sinopharm Chemical Reagent	CAS#: 60-00-4
EGTA	Sigma-Aldrich	CAS#: 67-42-5
Na_3_VO_4_	Sigma-Aldrich	CAS#: 13721-39-6
NaF	Sigma-Aldrich	CAS#: 7681-49-4
β-glycerophosphate disodium	Sigma-Aldrich	CAS#: 154804-51-0
DTT	Sigma-Aldrich	CAS#: 3483-12-3
Leupeptin	Sigma-Aldrich	CAS#: 103476-89-7
Antipain	Sigma-Aldrich	Cat#: 10791
Aprotinin	Roche	Cat#: 10981532001
PMSF	Sigma-Aldrich	CAS#: 9036-06-0
HCl	Sinopharm Chemical Reagent	CAS#: 7647-01-0
MgCl_2_	Sigma-Aldrich	CAS#: 7786-30-3
Histone type III-S	Sigma-Aldrich	CAS#: 9064-47-5
Triton X-100	Sigma-Aldrich	CAS#: 9036-19-5
β-mercaptoethanol	Sigma-Aldrich	CAS#: 60-24-2
Color PAGE gel rapid preparation kit	Epizyme	Cat#: PG-112
Blue Plus IV protein marker	TransGen	Cat#: DM131-01
Trichloroacetic acid (TCA)	Sigma-Aldrich	CAS#: 76-03-9
Sodium pyrophosphate (NaPPi, Na_4_P_2_O_7_)	Sigma-Aldrich	CAS#: 13472-36-1
PVDF transfer membrane, 0.45 μm	Invitrogen	Cat#: 88518
Methanol	Sinopharm Chemical Reagent	Cat#: 67-56-1
Western blot fast stripping buffer	Epizyme	Cat#: PS107
Goat anti-rabbit IgG (H+L)-HRP conjugate, EIA	Bio-Rad	Cat#: 172-1019
Anti-Myc tag (mouse monoclonal IgG1)	Merck/Millipore	Cat#: 05-724
Goat anti-mouse IgG (H+L)-HRP conjugate, EIA	Bio-Rad	Cat#: 172-1011
ACTIN (N) antibody, rabbit polyclonal	Abicode	Cat#: R3772-1P
Anti-GFP monoclonal antibody (JL-8)	Takara	Cat#: 632381
Anti-phospho-S175-SnRK2s antibody	ABclonal	Cat#: AP1481
Quick Start Bradford 1x dye reagent	Bio-Rad	Cat#: 5000205

**Experimental models: Organisms/strains**		

*Arabidopsis thaliana*: Col-0		N/A
*Arabidopsis thaliana*: *35Spro:SnRK2.1-YFP* in Col-0 background		N/A
*Arabidopsis thaliana*: *SnRK2.4pro:SnRK2.4-GFP* in *snrk2.1/4/5/7/8/9/10* mutant background		N/A

**Software and algorithms**		

Amersham Typhoon scanner	Amersham Typhoon	N/A
ImageQuant TL Control Centre	Cytiva	v.8.2.0

**Other**		

Percival incubator	Percival	CU36L5
Automatic grinding machine	Shanghai Jing Xin	JXFSTPRP-48
BioPhotometer Plus	Eppendorf	6132
Disposable cuvettes	Fisherbrand	T_70114955128
BAS-IP MS 2025 E phosphorimaging plate	Cytiva	28-9564-75
Amersham ERASER	Cytiva	29187190
Amersham Typhoon biomolecular imager	Cytiva	N/A
Gel and blot imaging systems	Cytiva	29187194
Model 583 and HydroTech pump gel drying complete systems	Bio-Rad	165-1790
Round disposable Petri dishes (130 × 130 mm)	Haimen Jiebo Co., Ltd (China)	EG1351
Square Petri dish (10 × 10 cm)	Haimen Jiebo Co., Ltd (China)	EG1350

## Materials and equipment

**Table:** Solid/liquid 1/2 MS plates, pH 5.7–5.8

Reagent	Final concentration	Amount
Murashige & Skoog (MS) Basal Salt Mixture	N/A	2.17 g
Sucrose	1%	10 g
Agar	0/1.2%	0/12 g
ddH_2_O	N/A	Up to 1 L
Total	N/A	1 L

***Note:*** Prepare fresh before use.

**Table:** Liquid 1/2 MS plates with mannitol, pH 5.7–5.8

Reagent	Final concentration	Amount
Murashige & Skoog (MS) Basal Salt Mixture	N/A	2.17 g
Sucrose	1%	10 g
Mannitol	300/600 mM	54.65/109.30 g
ddH_2_O	N/A	Up to 1 L
Total	N/A	1 L

***Note:*** Prepare fresh before use.

**Table:** Liquid 1/2 MS plates with ABA, pH 5.7–5.8

Reagent	Final concentration	Amount
Murashige & Skoog (MS) Basal Salt Mixture	N/A	2.17 g
Sucrose	1%	10 g
ABA	50 μM	50 μM
ddH_2_O	N/A	Up to 1 L
Total	N/A	1 L

***Note:*** Prepare fresh before use.

**Table:** PREMIX

Reagent	Final concentration	Amount
0.5 M HEPES, pH 7.5	100 mM	20 mL
0.5 M EDTA, pH 8.0	5 mM	1 mL
0.5 M EGTA, pH 7.0	5 mM	1 mL
0.5 M NaF	10 mM	2 mL
1 M β-Glycerophosphate	50 mM	5 mL
Glycerol	N/A	5 mL
ddH_2_O	N/A	Up to 100 mL
Total	N/A	100 mL

***Note:*** The buffer can be stored at 4^o^C for up to 6 months.

**Table:** Protein extraction buffer

Reagent	Final concentration	Amount
PREMIX	N/A	4.585 mL
0.5 M DTT	10 mM	100 μL
100 mM PMSF	1 mM	50 μL
200 mM Na_3_VO_4_	10 mM	250 μL
10 μg/μL antipain	5 μg/mL	5 μL
10 μg/μL leupeptin	5 μg/mL	5 μL
10 μg/μL aprotinin	5 μg/mL	5 μL
Total	N/A	5 mL

***Note:*** Prepare the buffer fresh immediately before use and keep it on ice.

**Table:** Running buffer, 10 ×

Reagent	Final concentration	Amount
Glycine	1.92 M	144 g
SDS	1% W/V	10 g
Tris	0.25 M	30 g
ddH_2_O	N/A	Up to 1 L
Total	N/A	1 L

***Note:*** Buffer can be stored at room temperature for up to 6 months.

**Table:** Transfer buffer, 5 ×

Reagent	Final concentration	Amount
Tris	0.125 M	15.1 g
Glycine	1.25 M	94 g
SDS	0.5% (W/V)	5 g
Methanol	20% (V/V)	200 mL
ddH_2_O	N/A	Up to 1 L
Total	N/A	1 L

***Note:*** Buffer can be stored at room temperature for up to 6 months.

**Table:** Transfer buffer, 1 ×

Reagent	Final concentration	Amount
Transfer buffer, 5 ×	20% (V/V)	200 mL
Methanol	20% (V/V)	200 mL
ddH_2_O	N/A	Up to 1 L
Total	N/A	1 L

***Note:*** Prepare fresh prior to use.

**Table:** TBST

Reagent	Final concentration	Amount
1 M Tris-HCl, pH7.5	25 mM	25 mL
5 M NaCl	30 mM	6 mL
Tween-20	0.06% (V/V)	600 μL
ddH_2_O	N/A	Up to 1 L
Total	N/A	1 L

***Note:*** Prepare fresh prior to use.

**Table:** Blocking buffer

Reagent	Final concentration	Amount
Skim milk powder	5% (W/V)	5 g
TBST	N/A	Up to 100 mL
Total	N/A	100 mL

***Note:*** Prepare fresh prior to use.

**Table:** Resolving gel for in-gel kinase assay

Reagent	Final concentration	Amount
ddH_2_O	N/A	1.3245 mL
1.5 M Tris–HCl, pH 8.8	0.37 M	0.875 mL
40% acrylamide/bis	N/A	0.8775 mL
Histone (10 mg/mL)	3.5 mg/mL	0.35 mL
10% ammonium persulfate (APs)	N/A	35 μL
10% SDS	N/A	35 μL
N, N, N′, N′-Tetramethyl Ethylene Diamine (TEMED)	N/A	3 μL
Total	N/A	3.5 mL

***Note:*** Prepare fresh prior to use.

**Table:** Stacking gel for in-gel kinase assay

Reagent	Final concentration	Amount
ddH_2_O	N/A	1.21 mL
1 M Tris–HCl, pH 6.8	0.25 M	0.5 mL
40% acrylamide/bis	N/A	0.248 mL
10% APs	N/A	20 μL
10% SDS	N/A	20 μL
TEMED	N/A	2 μL
Total	N/A	2 mL

***Note:*** Prepare fresh prior to use.

**Table:** SDS removal buffer

Reagent	Final concentration	Amount
1 M Tris–HCl, pH 7.5	25 mM	7.5 mL
0.5 M NaF	5 mM	3 mL
200 mM Na_3_VO_4_	66.67 μM	0.1 mL
BSA	0.05% (W/V)	0.15 g
Triton X-100	0.1% (V/V)	0.3 mL
ddH_2_O	N/A	Up to 300 mL
Total	N/A	300 mL

***Note:*** Prepare fresh prior to use.

**Table:** Renaturation buffer

Reagent	Final concentration	Amount
1 M Tris–HCl, pH 7.5	25 mM	7.5 mL
0.5 M NaF	5 mM	3 mL
200 mM Na_3_VO_4_	66.67 μM	0.1 mL
1 M DTT	0.5 mM	150 μL
ddH_2_O	N/A	Up to 300 mL
Total	N/A	300 mL

***Note:*** DTT should be added fresh immediately before use. Keep buffer on ice.

**Table:** Kinase reaction buffer I

Reagent	Final concentration	Amount
0.5 M HEPES, pH 7.5	40 mM	12 mL
1 M MgCl_2_	2 mM	2 mL
0.5 M EGTA, pH 7.0	2 mM	0.6 mL
200 mM Na_3_VO_4_	0.1 mM	75 μL
ddH_2_O	N/A	Up to 150 mL
1 M DTT	1 mM	150 μL
Total	N/A	1 L

***Note:*** DTT should be added fresh immediately before use. Keep buffer on ice.

**Table:** Wash solution

Reagent	Final concentration	Amount
TCA	0.306 M	25 g
NaPPi	37.6 mM	5 g
ddH_2_O	N/A	Up to 500 mL
Total	N/A	500 mL

***Note:*** Prepare fresh prior to use.

**Table:** Immunoprecipitation (IP) lysis buffer, 2 ×

Reagent	Final concentration	Amount
1 M Tris-HCl, pH 7.5	40 mM	0.4 mL
5 M NaCl	200 mM	0.4 mL
0.5 M EDTA, pH 8.0	2 mM	40 μL
0.25 M EGTA, pH 8.0	2 mM	80 μL
0.2 M Na_3_VO_4_	2 mM	100 μL
0.5 M NaF	2 mM	40 μL
1 M β-Glycerophosphate	20 mM	0.2 mL
10 mg/mL Leupeptin	4 μg/mL	4 μL
10 mg/mL Aprotinin	4 μg/mL	4 μL
10% Tween-20	0.2%	0.2 mL
ddH_2_O	N/A	Add to 10 mL
Total	N/A	10 mL

***Note:*** Prepare the buffer fresh immediately before use and keep it on ice.

**Table:** Kinase buffer, 5 ×

Reagent	Final concentration	Amount
1 M Tris-HCl, pH 7.5	125 mM	125 μL
1 M MgCl_2_	50 mM	50 μL
1 M DTT	5 mM	5 μL
ddH_2_O	N/A	Up to 1 mL

***Note:*** Prepare the buffer fresh immediately before use and keep it on ice.

**Table:** Reaction system

Reagent	Final concentration	Amount
Kinase buffer, 5 ×	N/A	4 μL
SnRK2 kinases	N/A	9 μL
Substrates (histones)	N/A	5 μL
ATP	N/A	2 μL
Total	N/A	20 μL

***Note:*** Prepare the buffer fresh immediately before use and keep it on ice.

## Step-by-step method details

### Preparation of plant materials

**Timing: 8–10 days**

This step applies to all four methods. It describes details that might influence the experimental results, including the sowing density and the duration of vernalization.

1. Sterilize the seeds using 5% sodium hypochlorite for 12 min in a 1.5 mL tube.
2. Wash the seeds four times with double-distilled water on a clean bench.
3. Plant the seeds evenly in a 10 cm × 10 cm square Petri dish that contains solid 1/2 MS, 1% sucrose, and 1.2% agar, pH 5.7–5.8 (Figure 1A).

**Figure 1:**
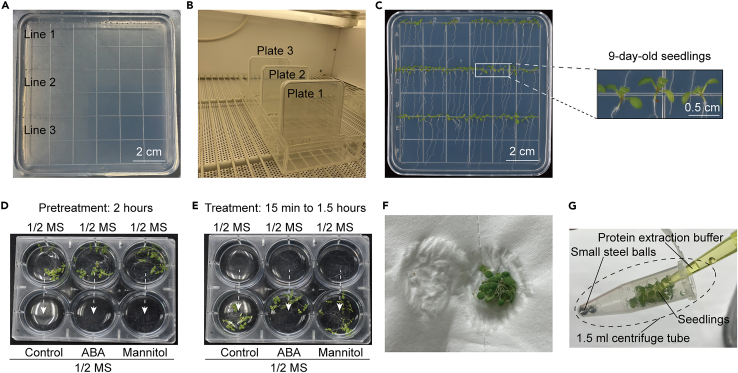
Sample preparation, stress treatment, and sample collection (A) Approximately 75 seeds are dotted uniformly onto the solid 1/2 MS medium. Scale bar: 2 cm. (B) Petri dishes are positioned upright with proper spacing in the incubator. (C) 9-day-old seedlings are used to evaluate SnRK2 activity in vivo. Scale bars: 2 cm (left) or 0.5 cm (right). (D) Gently transfer the seedlings to a six-well plate containing the liquid 1/2 MS medium and submerge them entirely for a two-hour pre-treatment. (E) Gently transfer the seedlings into the 1/2 liquid MS medium without or with ABA or mannitol, and incubate them for the indicated times. (F) Carefully remove the residual liquid medium from the seedlings with tissue paper after the treatments. (G) Protein extraction buffer is directly applied to seedlings to prevent ice immobilization of steel balls.
4. Seal the plates using micropore tape and stratify the seeds at 4°C for 48 h in the dark.
5. Grow the seedlings vertically in a Percival CU36L5 incubator at 22°C for 7 days under a 16-hour light and 8-hour dark photoperiod (Figures 1B and 1C).

**CRITICAL:** The seed planting density should be fixed to ensure the repeatability of the results. Typically, 75 seeds can be grown in three rows per Petri dish (Figure 1A). The intensity of light also affects SnRK2 activity. Therefore, the plant material prepared for the same experiment should be placed in the same location within the incubator, with a light intensity of 56–87.5 μmol m^−2^ s^−1^. Under high light conditions, cotyledons accumulate anthocyanin and are unsuitable for assessing SnRK2 activity.

### Treatment of seedlings with ABA, mannitol, or dehydration

**Timing: 6–8 h**

This step applies to all four methods and covers plant material selection, stress treatments, and sample collection.

6. Transfer the seedlings gently to a six-well plate containing 1/2 MS liquid medium and incubate at 22°C for 2 h (Figure 1D).

**CRITICAL:** Seedlings in different rows of the same dish experience varying light intensity. Select wild-type and mutant seedlings from the same row for consistency.

7. Prepare 1/2 MS liquid medium with 10 μM ABA and 0.3–0.8 M mannitol, pH 5.7–5.8.

**CRITICAL:** The 1/2 MS liquid medium for ABA and mannitol treatments should be prepared using the liquid medium for pretreatment in the same batch. This can enhance the reliability and repeatability of experimental results.

8. Prepare an appropriate volume of protein extraction buffer (50 μL for each sample).

***Note:*** The PREMIX reagent can be prepared in advance and stored at 4°C for an extended period. The protein extraction buffer with the complete formulation is light bright yellow and should be freshly prepared.

9. ABA, mannitol, and dehydration treatments (Figure 1E).
  a. For ABA and mannitol treatments, transfer the pretreated seedlings to the 1/2 MS liquid medium containing indicated concentrations of mannitol or ABA for the expected time points. The excess liquid medium needs to be removed by tissue paper.
  b. For dehydration treatment, place the pretreated seedlings on the absorbent paper for the expected time points.

**CRITICAL:** To improve the precision of treatments and control the duration of treatments accurately, the 1/2 MS liquid medium on seedlings after pretreatment needs to be removed by absorbent paper.

10. Use tissue paper to carefully blot the residual treatment solution on the seedlings, and then transfer the seedlings to a 1.5 mL centrifuge tube containing small steel balls (Figures 1F and 1G).
11. Use a pipette to carefully drop 50 μL protein extraction buffer onto the plant materials (Figure 1G). The samples were then quickly frozen in liquid nitrogen.

**CRITICAL:** Do not add the extraction buffer directly to the steel beads at the bottom of the centrifuge tube to prevent interference with the fragmentation of the sample during subsequent steps.

### Total protein extraction from plant materials

**Timing: 4 h**

This step applies to immunoblotting with anti-phospho-S175-SnRK2s antibody, in-gel kinase assay, and band shift assay. It describes the crucial operations to prevent protein degradation and maintain protein phosphorylation modifications during extraction.

12. Grind samples in the centrifuge tubes into powder using an automatic grinding machine.

**CRITICAL:** This step and all subsequent steps should be carried out on ice to avoid protein degradation.

13. Vortex thoroughly, and then leave the samples on ice for 10 min.
14. Centrifuge at 20,000 × g for 40 min at 4°C.
15. Transfer the supernatants to another 1.5 mL centrifuge tubes.
16. Add 3 μL of supernatant to 600 μL of 1 × Bradford reagent and measure the total protein concentrations using the BioPhotometer Plus.
17. Mix 80 μg of total proteins with 4 × SDS loading buffer in 40 μL of mixtures.
18. Boil the sample at 98°C for 3 min.

**CRITICAL:** The boiled samples can be directly used for electrophoresis or stored at −80°C after being quickly frozen in liquid nitrogen. However, the longer the storage time, the weaker the kinase signal becomes.

### SDS-gel preparation, electrophoresis, and blotting

**Timing: 3.5 h**

This step describes the parameters for SDS-page gel preparation, electrophoresis, and blotting in the immunoblotting assays with anti-phospho-S175-SnRK2s antibody and band shift assay.

19. Prepare a 10% SDS-PAGE gel (1.0 mm thickness, 15 wells) with the Color PAGE Gel Rapid Preparation Kit; the buffers are prefabricated and mixed according to the user guide in kit.
20. Load 15 μL of samples into each well. In the last well, load 5 μL of a protein molecular weight marker.

***Note:*** The protein molecular weight of SnRK2s is around 40 kDa. The protein marker should include appropriate molecular weights for subsequent experiments.

21. Perform electrophoresis at 80 V. When the target protein reaches the resolving gel, increase the voltage to 140 V.
22. Stop electrophoresis when the 25 kDa protein marker is about to run out of the gel.
23. Cut the gel according to the protein marker, and keep the gel portion between 25 kDa and 60 kDa.
24. Immerse the gel and thick filter papers in the transfer buffer.
25. Cut the PVDF membrane to an appropriate size. Immerse the PVDF membrane in methanol.
26. Transfer the protein onto the PVDF membrane using the Trans-Blot SD System (21 V for 60 min).

### Membrane blocking and antibody incubation

**Timing: 14 h**

This step describes the details of membrane blocking and antibody incubation in the immunoblotting experiments.

27. Block the PVDF membrane with the blocking buffer for 2 h.

***Note:*** From this step, the experiment should be performed on a horizontal shaker.

28. Transfer the PVDF membrane to fresh blocking buffer containing anti-phospho-S175-SnRK2s antibody (1:3000 dilution).
29. Incubate the PVDF membrane with primary antibody for 12–14 h at 4°C with gentle shaking.
30. Wash the PVDF membrane three times with TBST buffer (10 min per wash).
31. Transfer the PVDF membrane to the fresh blocking buffer containing secondary antibody (anti-rabbit, 1:5000 dilution).
32. Wash the PVDF membrane three times with TBST buffer (10 min per wash).

### Signal detection

**Timing: 0.5 h**

This step applies to the immunoblotting assay with anti-phospho-S175-SnRK2s antibody and band shift assay.

33. Prepare the chemiluminescent substrate solution according to the manufacturer’s instructions.
34. Incubate the PVDF membrane with the chemiluminescent substrate solution.

**CRITICAL:** The signal should be detected immediately after applying the chemiluminescent substrate solution to the PVDF membrane.

35. Acquire images using a chemiluminescence imaging system.

### Antibody stripping and reuse of the PVDF membrane

**Timing: 1.5 h**

This step describes the stripping of the primary antibody and the re-incubation method for another primary antibody in the immunoblotting experiments.

36. Wash the PVDF membrane three times with TBST buffer (10 min per wash).
37. Strip the anti-phospho-S175-SnRK2s or anti-GFP antibody from the PVDF membrane using Western Blot Fast Stripping Buffer for 20 min.
38. Wash the PVDF membrane three times with TBST buffer (10 min per wash).

***Note:*** The PVDF membrane can be re-blocked, and the anti-Actin antibody can be hybridized again. The following steps are the same as above (Steps 27–35).

### SDS-gel preparation and electrophoresis for in-gel kinase assay

**Timing: 3.5 h**

This step applies to in-gel kinase assay and describes its preparation, the selection of protein markers, and the parameters and duration of electrophoresis.

39. Prepare the resolving gel (with a 0.75 mm space and 15 wells) for the in-gel kinase assay, following the specifications above (requiring 3.5 mL of the resolving gel).

***Note:*** The thickness of the SDS-PAGE gel is crucial for this experiment. The most suitable thickness is 0.75 mm.

40. Cover the gel with ethanol. Allow the gel to polymerize at 22°C for 1 h.

**Figure 2:**
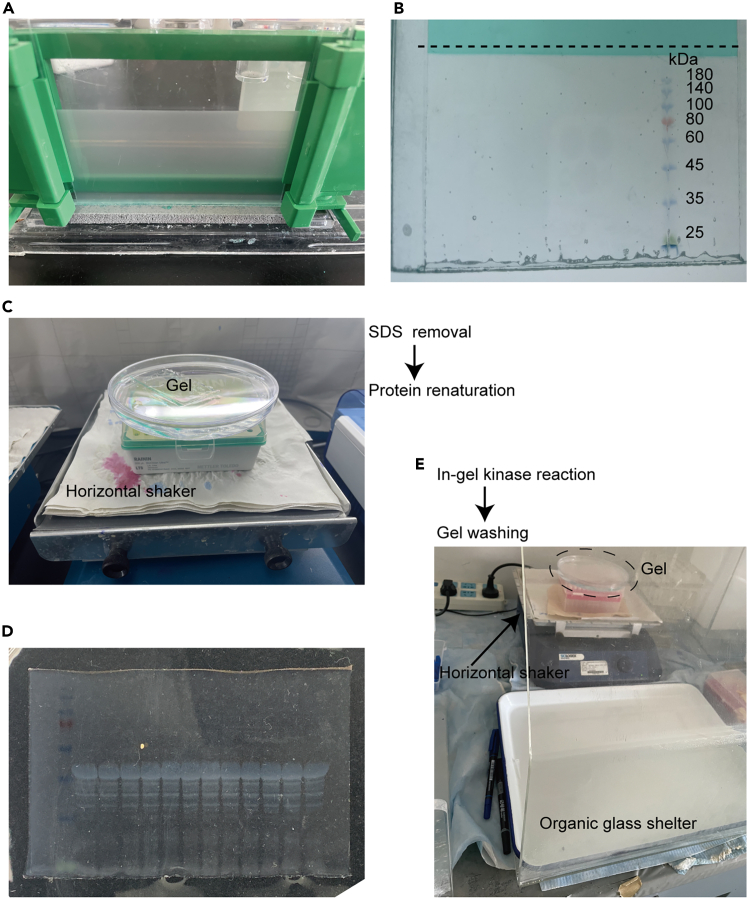
Crucial steps for the in-gel kinase assay (A) The freshly prepared lower-layer gel appears as a translucent milky-white colloid. (B) The 25 kDa marker band is positioned at the bottom of the gel. Carefully cut the gel along the dotted line and preserve the lower gel. (C) Completely immerse the gel in the SDS removal buffer to facilitate the SDS removal and protein renaturation. (D) During the process of SDS removal, the proteins embedded in the gel can be gradually observed. (E) The in-gel kinase reaction and gel-washing processes is carried out with proper protection against radioactive isotopes.

***Note:*** A proper polymerized lower-layer gel should be a translucent milky-white colloid (Figure 2A).

41. Pour off the ethanol and prepare the 0.75 mm stacking gel for the in-gel kinase assay as indicated in the table above (1.5 mL is required). Insert a 15-well comb. Let the gel polymerize at 22°C for an additional 1 h.
42. Assemble the gel into the electrophoresis tank using the fresh running buffer.
43. Load 13 μL of the sample into each well. One well should be reserved for 5 μL of the protein marker.

**CRITICAL:** The protein marker used in this experiment must not contain protein kinases. Otherwise, these protein kinases may retain activity during subsequent experiments and interfere with the results.

44. Perform electrophoresis at 80 V. After about half an hour, adjust the voltage to 120 V once proteins migrated into the separating gel.
45. Stop the electrophoresis when the 25 kDa protein marker band reaches the bottom edge of the gel (Figure 2B).

**CRITICAL:** The complete resolving gel and approximately 2 mm of the stacking gel should be retained. Avoid trimming the resolving gel as this may cause breakage during subsequent steps.

### SDS removal and protein renaturation

**Timing: 16 h**

This step applies to in-gel kinase assay and describes the methods for removing SDS from the gel and subsequent protein renaturation.

46. Prepare 300 mL of SDS removal buffer.
47. Immerse the gel in a 130 mm-diameter Petri dish containing 100 mL of SDS removal buffer.
48. Place it on a horizontal shaker at low speed for 20 min (Figure 2C).

***Note:*** From this step, the experiment should be performed on a horizontal shaker at low speed.

49. Repeat Steps 47 and 48 twice, using another 130 mm-diameter Petri dish containing 100 mL of fresh SDS removal buffer each time.

***Note:*** During the process of SDS removal, protein bands can gradually be seen clearly in the gel (Figure 2D).

50. Prepare 300 mL of renaturation buffer. Add DTT immediately before use.
51. Transfer the gel to another 130 mm-diameter Petri dish containing 100 mL of renaturation buffer, and then incubate on the horizontal shaker at low speed for 1 h (Figure 2C).
52. Transfer the gel to another 130 mm-diameter Petri dish containing 100 mL of renaturation buffer, and then incubate on the horizontal shaker at low speed 12–14 h.

**CRITICAL:** The in-gel kinase assay takes two days. This step can usually be carried out in a 4°C environment, such as cold storage. Additionally, it is necessary to prevent the buffer evaporation due to prolonged shaking.

53. Repeat Step 51.

### In-gel kinase reaction

**Timing: 2.5 h**

This step describes the detailed parameters of the kinase reaction.

54. Prepare 150 mL of kinase reaction buffer I. Add DTT to 1 mM immediately before use.
55. Transfer the gel to a 130 mm-diameter Petri dish that contains 100 mL of kinase reaction buffer I for pre-reaction, and then incubate on a horizontal shaker at a low speed for 30 min.
56. Transfer the gel to a 130 mm-diameter Petri dish containing 40 mL of kinase reaction buffer I, then add 4 μL of [γ-^32^P] ATP. Shake the gel horizontally at a low speed for 5 min.

***Note:*** Radioactive isotopes are used in this step. Therefore, this step and subsequent experiments should be performed with appropriate radiation protection (Figure 2E).

57. Add 6 μL of 1 mM ATP to the kinase reaction buffer I. Gently continue gentle rotation at 22°C for 2 h.

***Note:*** The optimal reaction temperature is 30°C. If the room temperature is too low, consider using temperature control equipment such as an air conditioner.

### Gel washing and drying

**Timing: 6–7 h**

This step applies to in-gel kinase assay and describes the methods for washing and drying the gel after the kinase reaction.

58. Prepare 500 mL of washing solution.

***Note:*** TCA is highly corrosive. Always wear appropriate protective gloves when preparing and handling the washing solution.

59. Transfer the gel to another 130 mm-diameter Petri dish containing 100 mL of washing solution, and incubate on a horizontal shaker at low speed for 1 h.

**CRITICAL:** The washing solution makes the gel fragile, so transfer the gel gently.

**Figure 3:**
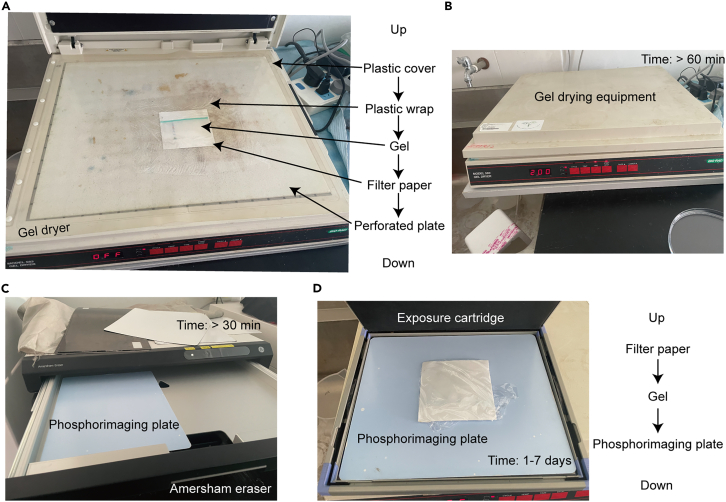
Critical steps for gel drying and signal acquisition (A and B) The proper stacking sequence of different items during the gel drying (A) and the corresponding time setting (B). (C) The phosphorimaging plate is placed with the front side facing upward to remove background signals. (D) The dried gel is positioned to face the front surface of the phosphorimaging plate.
63. Dry the gel under vacuum at 80°C for 1 h using the gel dryer coupled with a HydroTech Vacuum Pump (Figure 3B).
64. Utilize the Amersham eraser to expose the phosphorimaging plate for 30 min (Figure 3C).
65. Encase the gel that is adhered to the filter paper with plastic wrap, and then position the gel on the front surface of the phosphorimaging plate (Figure 3D).

***Note:*** This operation should be conducted within an exposure cartridge capable of shielding radioactive isotopes.

60. Repeat Step 59 four times.
61. Gently mount the protein gel onto filter paper and transfer the assembly to the perforated plate of the gel dryer.
62. Thoroughly cover the gel and the filter paper with plastic wrap, then secure with the gel dryer’s plastic cover (Figure 3A).

### Signal acquisition and imaging

**Timing: 2–7 days**

This step applies to in-gel kinase assay and describes the method for observing the experimental results.

66. Expose the dried gel onto the storage phosphor screens for a sufficient time (2–7 days).
67. Attach the phosphorimaging plate firmly to the slip sheet and record its position (Figure 4A).

**Figure 4:**
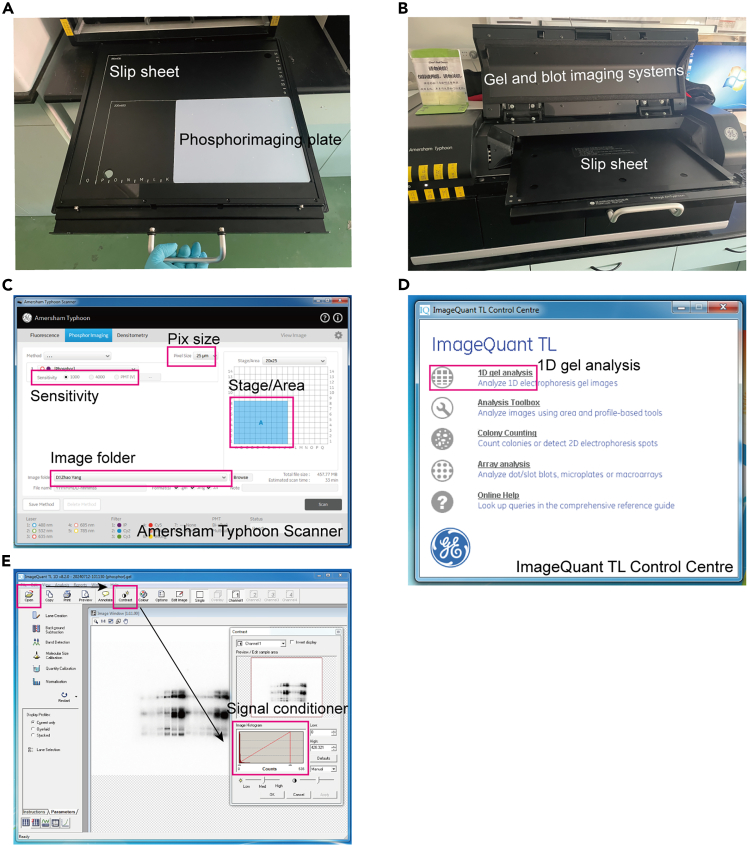
Signal scanning and imaging (A and B) The phosphorimaging plate is carefully attached to the slip sheet with its backside facing toward the sheet (A) and then placed in the instrument according to the operation manual (B). (C) Parameters for the Amersham Typhoon Scanner for accurate signal scanning. (D and E) The proper operation using the ImageQuant TL Control Centre software (D) and fine-tuning of signal strength (E).
68. Position the slip sheet within the Gel and blot imaging systems per the operation manual (Figure 4B).
69. Acquire and save the image using the Gel and blot imaging systems (Figure 4C).
70. Employ 1D gel analysis of the ImageQuant TL Control Centre to precisely regulate the signal intensity and save the image (Figures 4D and 4E).

### Band shift of phosphorylated SnRK2s

**Timing: 6–7 h**

This step describes the method for detecting phosphorylated SnRK2.1 and SnRK2.4. A small portion of SnRK2s can be phosphorylated *in planta* under stress conditions. The molecular weight of phosphorylated SnRK2s is larger than that of unphosphorylated SnRK2s, which may lead to a shift of the protein band during electrophoresis. Therefore, immunoblotting with a corresponding anti-GFP antibody is used for detecting phosphorylated SnRK2.4, using the *SnRK2.4pro:SnRK2.4-GFP* transgenic lines in the *snrk2.1/4/5/7/8/9/10* mutant background.^3^

71. The steps of protein extraction and western blot are described in Steps 12–27.
72. Transfer the PVDF membrane to a fresh blocking buffer and add anti-GFP and anti-Actin antibodies at a ratio of 1:3000 or 1:10000, respectively.
73. Incubate the PVDF membrane at 22°C for 2 h (or 12 - 14 h at 4°C) at low speed.
74. Wash the PVDF membrane three times with TBST buffer for 10 min each time.
75. Transfer the PVDF membrane to a fresh blocking buffer and add the corresponding secondary antibody at a ratio of 1:10000, and incubate the PVDF membrane at 22°C for 1 h.
76. Wash the PVDF membrane three times with TBST buffer for 10 min each time.
77. Prepare the chemiluminescent substrate solution according to the instructions.
78. Incubate the PVDF membrane in the chemiluminescent substrate solution.
79. Acquire images using the chemiluminescence imaging system.

**CRITICAL:** This assay detects the ratio of phosphorylated SnRK2s to the unphosphorylated SnRK2s. Since only a small portion of SnRK2s can be phosphorylated *in planta* under stress conditions, the total protein abundance of SnRK2s in transgenic lines determines the performance of this experiment. Due to notably high protein levels, the *SnRK2* overexpression lines are unsuitable for band shift assays.

***Note:*** If the signal is weak, prolong the exposure time or increase the antibody concentration.

### Precipitation of protein from *Arabidopsis* seedlings

**Timing: 1 day**

This step applies to the immunoprecipitated kinase assay. It describes the immunoprecipitation of SnRK2s and the crucial procedures to maintain phosphorylation modification on SnRK2s during extraction. Transgenic lines expressing tagged *SnRK2* (for example, *SnRK2-YFP*) are used.

80. Plant material is prepared and treated as Steps 1-9.
81. The samples are blotted dry (about 0.5 g) and quickly frozen in liquid nitrogen.
82. Place samples into mortars (precooled in liquid nitrogen) and grind thoroughly.
83. Add 1 mL of immunoprecipitation (IP) lysis buffer, mix thoroughly, and incubate the lysates on ice for 30 min.
84. Transfer the samples into 2 mL tubes and centrifuge at 14,500 × g for 30 min at 4°C. Meanwhile, wash GFP magnetic beads three times with 1 × IP lysis buffer using a magnetic grate.

***Note:*** About 1 mL lysis buffer is added to each sample, which can be increased or decreased according to the amount of sample for grinding.

85. Transfer the supernatants into another 1.5 mL tubes and incubate with GFP magnetic beads for 3 h at 4°C on a rotating wheel.
86. Centrifuge and remove the supernatants. Wash the beads three times with 1 × IP lysis buffer using the magnetic rack. Then store at −80°C.

***Note:*** Beads should be aliquoted before freezing to avoid repeated freezing and thawing when stored at −80°C.

### Immunoprecipitated kinase assay

**Timing: 1 day**

This step describes a kinase assay with the immunoprecipitated SnRK2s using substrates (e.g., histones).

87. Prepare samples for the kinase assay.
  a. Thaw SnRK2-binding GFP magnetic beads on ice.
  b. Prepare 1 × kinase buffer.
  c. Balance the thawed GFP magnetic beads with 1 × kinase buffer 3 times for 5-10 min each time.
  d. After removing the supernatants, only a small amount of buffer remains.

***Note:*** All operations must be performed on ice to avoid protein degradation and maintain phosphorylation modifications.

***Note:*** If this step needs to be paused, the sample can be kept on ice for up to one week.

88. Set up 20 μL reaction system for YFP-tagged SnRK2s purified from transgenic lines treated without or with mannitol.
89. Add [γ-^32^P] ATP in the 20 μL reactions and incubate at 30°C for 2 h.
  a. Place the reactions on ice.
  b. Prepare an ATP mixture containing 2 μL [γ-^32^P] ATP and unlabeled ATP solution (to a final concentration of 1.25 μM).
  c. Add 2 μL [γ-^32^P] ATP mixtures to each reaction at the bottom of the tube and mix well.
  d. Incubate in a metal bath at 30°C for 2 h.

***Note:*** To reduce the interference of low SnRK2 protein abundance, adding 9 μL suspensions of the SnRK2-binding beads to the 20 μL reaction is optimal.

***Note:*** Tubes should have explosion-proof clips to prevent sample explosion during heating.

90. Stop the reaction by adding 5 μL of 5 × loading buffer. After securing with explosion-proof clips, heat the mixtures at 98°C for 5 min and centrifuge.
91. Prepare the SDS-page gel and perform electrophoresis as described in Steps 19–22.
92. Stain the gels for 30 min with Coomassie Brilliant Blue (R250).
93. Wash to remove unbound Coomassie Brilliant Blue dye from the gel and capture images.
94. Dry the gel under vacuum at 80°C for 1 h on filter paper as described in Steps 61–63.

### Signal acquisition and imaging

**Timing: 6–7 h**

This step is the same as the signal acquisition described in Steps 64–70. Since the signal is stronger than in in-gel kinase assays, exposure of the storage phosphor screens for 6–7 h or 12–14 h is sufficient.

**CRITICAL:** If the signal is weak, reaction conditions can be adjusted (e.g., concentrations of kinases and substrates), and the exposure time can be increased.

***Note:*** The molecular weights of kinases and substrates should be different to avoid interference between autophosphorylation and transphosphorylation.

***Note:*** Kinase activity decreases with longer storage time, so newly purified protein kinases are recommended for each experiment.

## Expected outcomes

**Figure 5:**
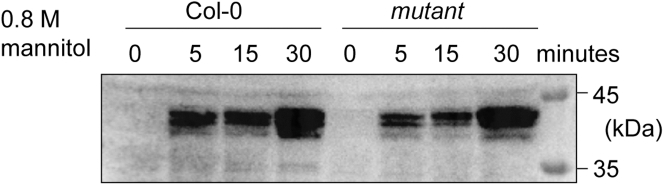
Immunoblotting results with anti-phospho-S175-SnRK2s antibodies The Col-0 wild-type and mutant seedlings were treated with 0.8 M mannitol for the indicated times. Phosphorylation of serine residues in the activation loop of multiple SnRK2s corresponding to Ser175 in SnRK2.6 was detected using anti-phospho-S175-SnRK2s antibodies.

**Figure 6:**
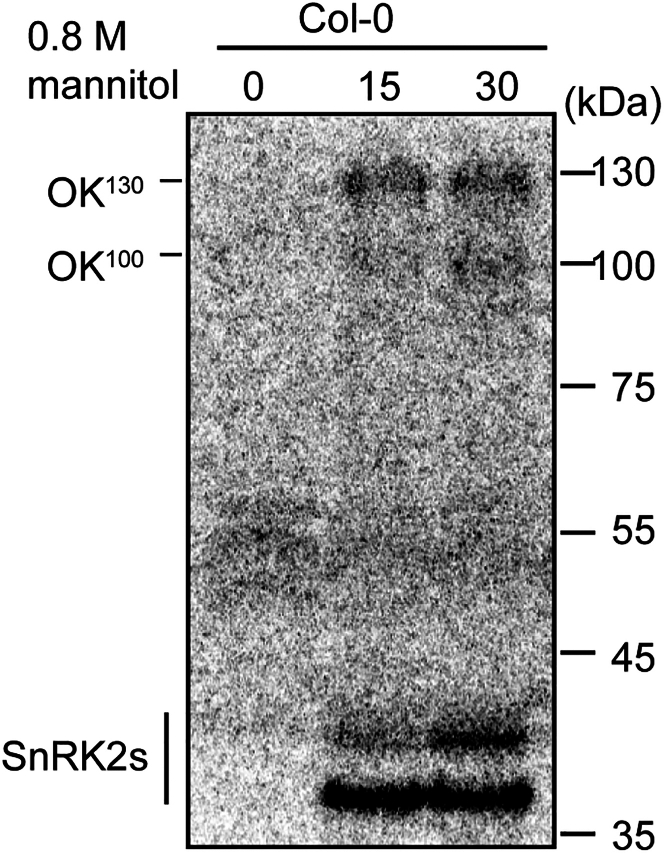
In-gel kinase results detecting SnRK2 activation The Col-0 wild-type seedlings were treated with 0.8 M mannitol for the indicated times. The SnRK2 bands appear around 40 kDa, while the RAF bands appear around 100 and 130 kDa. The B4 RAFs are also named osmotic stress-activated (OK)^130^, while the B2 and B3 RAFs are also named OK^100^.

**Figure 7:**
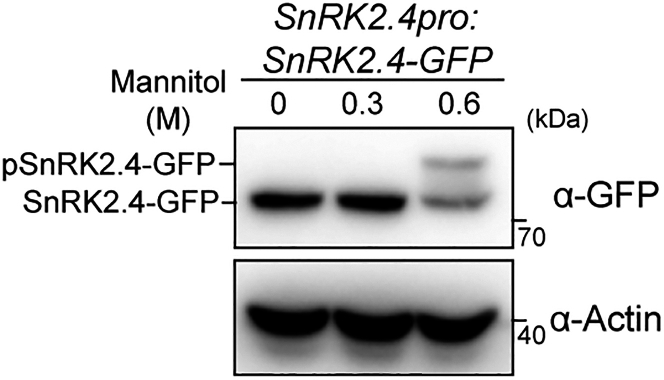
Band shift results representing SnRK2.4 activation by severe osmotic stress Band shift of SnRK2.4-GFP in 7-day-old *SnRK2.4pro:SnRK2.4-GFP* transgenic seedlings in the *snrk2.1/4/5/7/8/9/10* septuple mutant background, after treatments with 0.3 M or 0.6 M mannitol for 30 min. SnRK2.4-GFP was detected by an anti-GFP antibody (top). Actin served as the loading control (bottom).

**Figure 8:**
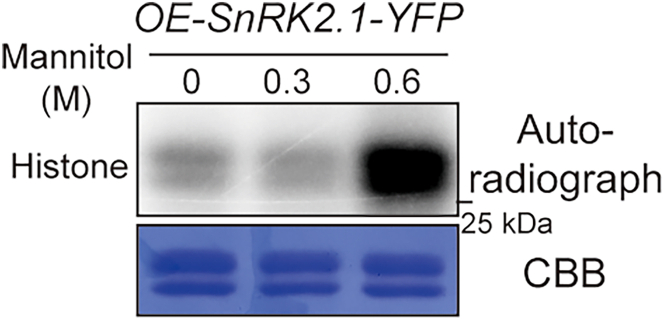
Immunoprecipitated kinase assay showing SnRK2.1 activation by severe osmotic stress Kinase activity of YFP-tagged SnRK2.1 immunoprecipitated from the *SnRK2.1-YFP* overexpression lines after 30 min treatments with different concentrations of mannitol (0.3 and 0.6 M). Autoradiography (top) and Coomassie staining (bottom) exhibit phosphorylation and loading of histone, respectively.

The immunoblotting assay with anti-phospho-S175-SnRK2s antibody could detect phosphorylated endogenous SnRK2s and exogenous YFP-tagged SnRK2s.^3^ The phosphorylated endogenous SnRK2s are around 40 kDa, and these bands may merge in different extents (Figure 5).^3^ The in-gel kinase assay could detect activated SnRK2 and RAF protein kinases that phosphorylate histones and ABF fragments.^1^ The SnRK2 bands range from 37 to 42 kDa and merge to different extents in different experiments, while the RAF bands are around 100 and 130 kDa (Figure 6). In the band shift assay, phosphorylation and upshift of the SnRK2.4-GFP band could be triggered by treatment with 0.6 M mannitol but not 0.3 M mannitol (Figure 7). In the immunoprecipitated kinase assay, histones could be phosphorylated by SnRK2.1-YFP activated by 0.6 M mannitol, whereas no phosphorylation was observed with immunoprecipitated SnRK2.1-YFP after 0.3 M mannitol treatment (Figure 8).

## Limitations

The immunoblotting assay with anti-phospho-S175-SnRK2s antibody represents the phosphorylation of serine residues in the activation loop of multiple SnRK2s corresponding to Ser175 in SnRK2.6. The phosphorylation level of this conserved serine reflects SnRK2 kinase activity. However, different batches of polyclonal antibodies exhibit preferences for different SnRK2s.^3^ Therefore, it is crucial to generate monoclonal antibodies for anti-phospho-S175-SnRK2s. Although in-gel kinase assay represents an unbiased method for evaluating endogenous SnRK2s, it requires the radioactive isotope labeled [γ-^32^P] ATP and specialized experimental equipment and facilities. Additionally, the experimental procedure is much more complicated than that of the immunoblotting assay. For band shift and immunoprecipitated kinase assays, transgenic lines expressing tagged SnRK2s are required.

## Troubleshooting

### Problem 1

Significant differences in total protein concentrations among different samples.

### Potential solution

The exact same number of seedlings grown on the same 1/2 MS medium in the duplicate rows should be used for protein extraction. Besides, it is optimal to handle no more than 12 samples at once to avoid extended extraction that may increase the risk of protein degradation (related to Steps 12 and 16).

### Problem 2

The signal of phosphorylated SnRK2s in the immunoblotting assay is too weak or undetectable.

### Potential solution

This may be caused by protein degradation or dephosphorylation of the conserved serine in SnRK2s during the protein extraction. The whole extraction process should be performed on ice, with the addition of phosphatase inhibitors. Besides, excessive plant materials should be avoided during the protein extraction procedure. Moreover, the total proteins should be freshly prepared. Although boiled samples could be stored at −80°C, the storage time is preferably within two weeks. Finally, increasing the antibody concentration (up to 1:1000), extending the exposure time, and using a more sensitive chemiluminescent substrate solution (such as a hypersensitive type) may enhance the signal to some extent (related to Steps 12, 13, 18, 28 and 33).

### Problem 3

The signal of SnRK2s in the in-gel kinase assay is too weak or undetectable.

### Potential solution

The potential solution could be the same as for problem 2. This problem can also be caused by excessive gel thickness. For an in-gel kinase assay, the phosphorylation reaction occurs within the gel, and excessive thickness will interfere with the contact between the kinase reaction buffer and proteins embedded in the gel (e.g., the endogenous protein kinases and the histone substrates). Hence, the optimal thickness of the gel is 0.75 mm (related to Step 39).

This problem can also be caused by the decay of the radioactive isotope that labels [γ-^32^P] ATP (related to Step 56). The in-gel kinase assay is optimal when performed with fresh [γ-^32^P] ATP (within 7 days of production). The usage of decayed isotopes should be doubled appropriately (within 2 weeks of production).

### Problem 4

The protein abundance of the immunoprecipitated SnRK2s is very low.

### Potential solution

This may be caused by the lower expression level of SnRK2s in transgenic lines, weak affinity of the beads, or an insufficient lysis process. Therefore, this experiment requires transgenic lines with enough SnRK2s and effective affinity beads. The extraction of total proteins should use a freshly prepared lysis buffer (related to Steps 80 to 83).

### Problem 5

The immunoblotting results have a high background.

### Potential solution

This may be caused by insufficient membrane blocking or washing. Therefore, a prolonged blocking process or milk replacement with bovine serum albumin (BSA) may resolve this problem (related to Step 27). A prolonged washing process may also help.

## Resource availability

### Lead contact

Further information and requests for resources and reagents should be directed to and will be fulfilled by the lead contact, Yang Zhao (yangzhao@psc.ac.cn).

### Technical contact

Technical questions on executing this protocol should be directed to and will be answered by the technical contact, Yang Zhao (yangzhao@psc.ac.cn).

### Materials availability

All the non-commercial materials described in this study are available upon request.

### Data and code availability

Original data have been deposited to Mendeley data: https://doi.org/10.17632/hk5bs8yys3.1. This study did not generate/analyze datasets.

## Acknowledgments

We thank members of the Zhao Lab for helpful discussions. This work was supported by the Project of Stable Support for Youth Teams in Basic Research Field of the Chinese Academy of Sciences (YSBR-119), the Strategic Priority Research Program of the Chinese Academy of Sciences (grant no. XDB063020102), and the Shanghai Center for Plant Stress Biology from the Chinese Academy of Sciences.

## Author contributions

Q.L., X.Y., and Y.Z. wrote the manuscript; Q.L. and X.Y. prepared figures and tables.

## Declaration of interests

The authors declare no competing interests.
